# Evaluation of molecular receptors status in breast cancer using an mpMRI-based feature fusion radiomics model: mimicking radiologists’ diagnosis

**DOI:** 10.3389/fonc.2023.1219071

**Published:** 2023-11-21

**Authors:** Shengsheng Lai, Fangrong Liang, Wanli Zhang, Yue Zhao, Jiamin Li, Yandong Zhao, Yongzhou Xu, Wenshuang Ding, Jie Zhan, Xin Zhen, Ruimeng Yang

**Affiliations:** ^1^ School of Medical Equipment, Guangdong Food and Drug Vocational College, Guangzhou, Guangdong, China; ^2^ Department of Radiology, The Second Affiliated Hospital, School of Medicine, South China University of Technology, Guangzhou, Guangdong, China; ^3^ Department of Radiology, Guangzhou First People’s Hospital, Guangzhou, Guangdong, China; ^4^ Department of Clinical & Technique Support, Philips Healthcare, Guangzhou, Guangdong, China; ^5^ Department of Pathology, Guangzhou First People’s Hospital, Guangzhou, Guangdong, China; ^6^ Department of Radiology, The First Affiliated Hospital of Nanchang University, Nanchang, Jiangxi, China; ^7^ School of Biomedical Engineering, Southern Medical University, Guangzhou, Guangdong, China

**Keywords:** breast cancer, magnetic resonance imaging, molecular receptor, radiomics, classification

## Abstract

**Objective:**

To investigate the performance of a novel feature fusion radiomics (R_FF_) model that incorporates features from multiparametric MRIs (mpMRI) in distinguishing different statuses of molecular receptors in breast cancer (BC) preoperatively.

**Methods:**

460 patients with 466 pathology-confirmed BCs who underwent breast mpMRI at 1.5T in our center were retrospectively included hormone receptor (HR) positive (HR+) (n=336) and HR negative (HR-) (n=130). The HR- patients were further categorized into human epidermal growth factor receptor 2 (HER-2) enriched BC (HEBC) (n=76) and triple negative BC (TNBC) (n=54). All lesions were divided into a training/validation cohort (n=337) and a test cohort (n=129). Volumes of interest (VOIs) delineation, followed by radiomics feature extraction, was performed on T2WI, DWI_600_ (b=600 s/mm^2^), DWI_800_ (b=800 s/mm^2^), ADC map, and DCE_1-6_ (six continuous DCE-MRI) images of each lesion. Simulating a radiologist’s work pattern, 150 classification base models were constructed and analyzed to determine the top four optimum sequences for classifying HR+ *vs*. HR-, TNBC *vs*. HEBC, TNBC *vs*. non-TNBC in a random selected training cohort (n=337). Building upon these findings, the optimal single sequence models (Rss) and combined sequences models (R_FF_) were developed. The AUC, sensitivity, accuracy and specificity of each model for subtype differentiation were evaluated. The paired samples Wilcoxon signed rank test was used for performance comparison.

**Results:**

During the three classification tasks, the optimal single sequence for classifying HR+ *vs*. HR- was DWI_600_, while the ADC map, derived from DWI_800_ performed the best in distinguishing TNBC *vs*. HEBC, as well as identifying TNBC *vs*. non-TNBC, with corresponding training AUC values of 0.787, 0.788, and 0.809, respectively. Furthermore, the integration of the top four sequences in R_FF_ models yielded improved performance, achieving AUC values of 0.809, 0.805 and 0.847, respectively. Consistent results was observed in both the training/validation and testing cohorts, with AUC values of 0.778, 0.787, 0.818 and 0.726, 0.773, 0.773, respectively (all *p* < 0.05 except HR+ *vs*. HR-).

**Conclusion:**

The R_FF_ model, integrating mpMRI radiomics features, demonstrated promising ability to mimic radiologists’ diagnosis for preoperative identification of molecular receptors of BC.

## Introduction

Breast cancer (BC) exhibits significant heterogeneity at both intra- and inter-tumor levels. Different molecular receptor statuses are associated with varying prognoses, treatment responses and survival outcomes (1, 2). Profiling of gene expression has identified the four main intrinsic molecular subtypes of BC, including luminal A, luminal B, human epidermal growth factor receptor 2-enriched (HER-2), and triple negative (TN), each of which exhibits distinct molecular receptor statuses and therefore requires tailored therapeutic approach, such as endocrine therapy or neoadjuvant systemic therapy (NST) (3–5).

Currently, molecular receptor status is mainly determined by gene expression profiling or immunohistochemical (IHC) surrogates from invasive tissue biopsy or surgical specimens in clinical practice. However, due to tumor heterogeneity, a single tissue biopsy is insufficient to capture the global genetic, epigenetic, and/or phenotypic characteristics of a breast tumor, leading to inevitable selection bias (1, 2). In addition, as the tumor biology evolves and continuous treatments are administrated, the receptor status and molecular subtypes of BC may change, posing challenges in accurately reflecting the true state of the lesions (5). Therefore, there is a need to develop an effective method for precise assessment of the whole-tumor’s histological characteristics, and for spatial-temporal monitoring of the dynamic tumor biological behavior during treatment.

MRI-based radiomics, which uses data-mining algorithms or statistical analysis tools on high-throughput imaging features to obtain predictive or prognostic information, has shown promising potentials as an alternative tool for the assessment of BC’s molecular receptors status (6–8). Multiparametric magnetic resonance imaging (mpMRI), which combines morphological (T2 weighted-imaging [T2WI]), functional (diffusion-weighted imaging [DWI]) and kinetic (dynamic contrast-enhanced [DCE]) information, has further demonstrated great promise for preoperative identification of different molecular receptor statuses of BC (8–10). However, previous investigations mainly selected only one or two single MRI sequence-derived images (e.g., T2WI, DWI-derived apparent diffusion coefficient [ADC] maps, or the early phase of DCE-MRI) for analysis (7, 11–14), which deviates from the real clinical scenario where radiologists routinely go through all acquired MRI images to make a final diagnosis. Without a comprehensive consideration of the various contributions from different MRI sequences, it may result in subjectivity and an insufficient assessment.

Herein, we hypothesize that a mpMRI-based radiomics method has the potential to provide accurate prediction of molecular subtypes and receptor status of BC. The aim of this study is to develop a novel feature fusion radiomics (R_FF_) model that incorporates radiomics features extracted from optimally performed mpMRIs to mimic the routine diagnostic practices of radiologists and preoperatively identify different molecular receptor statuses in BC.

## Materials and methods

### Patient cohort

**Figure 1:**
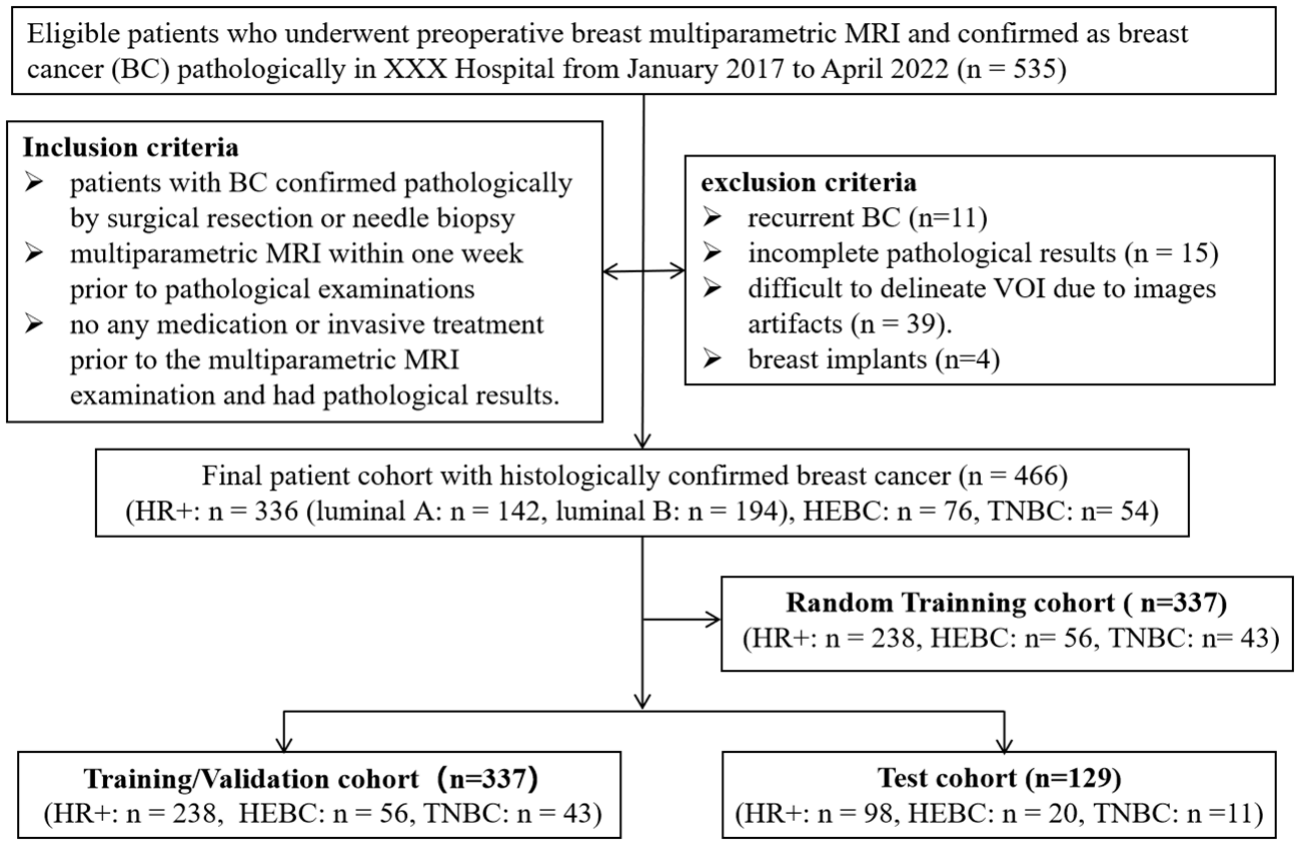
Flow chart of the study’s population with inclusion and exclusion criteria. BC, breast cancer; HEBC, human epidermal growth factor receptor 2 enriched BC. TNBC, triple-negative breast cancer. “n=466” represented the total lesion number.

This study was approved by the Ethics Committee of the Second Affiliated Hospital of South China University of Technology (Guangzhou First People’s Hospital) Hospital, with informed consent being waived due to the retrospective nature of this study. A total of 535 patients who underwent breast mpMRI for preoperative assessment at our hospital between January 2017 and April 2022 were included. The inclusion criteria were as follows: (1) histopathological confirmation of BC by surgical resection or needle biopsy; (2) patients who underwent a routine mpMRI including T1WI, T2WI, DWI (with b values of 0 s/mm^2^, 600 s/mm^2^ and 800 s/mm^2^), DWI-derived ADC map and DCE-MRI (with 6 continuous enhancing phases) within one week prior to pathological examinations; (3) no additional therapy prior to MRI. The exclusion criteria were: (1) recurrent BC (n=11); (2) incomplete pathological results, such as those lacking IHC results and Ki-67 scores, or unclear histological types (n=15); (3) cases with Volumes of interest (VOI) that were difficult to delineate due to images artifacts (n=39). (4) patients with breast implants (n=4). In cases of multicentric or multifocal tumors, only the largest malignant lesion was selected. For bilateral disease, the largest lesions of both breasts were selected according to pathological results. Finally, 460 patients with 466 lesions were enrolled in this study. The lesions were categorized into HR+ (n=336) and HR- groups (n=130), with the HR- group further divided into HEBC (n=76) and TNBC (n=54) subgroups. Based on sample size calculations (15, 16), a required sample size of 210 (42 cases of TNBC and 168 cases of non-TNBC) was sufficient to detect differences between various molecular subtypes of BC with a power of 95%. **Appendix 1** showed the detailed information on the sample size calculation process. All lesions were divided randomly into a training/validation cohort (n=337) and a test cohort (n=129) at a ratio of ~3:1, in which a random selected training cohort (n=337) was established to determine the optimal single MR sequence for subsequent experiments, as shown in **Figure 1**.

### MRI acquisition

All preoperative routine mpMRI examinations were performed on a 1.5-T MRI system (uMR 560, United Imaging) using a dedicated 4-channel SENSE breast coil. The standard examinations included T1W, T2W, DWIs (with three b values of b=0 s/mm^2^, b=600 s/mm^2^ (DWI_600_) and b=800 s/mm^2^ (DWI_800_)) and six continuous DCE-MRI (DCE_1-6_) scans with ~62 seconds per phase. ADC maps were derived from DWI using the b=0 s/mm^2^ and b=800 s/mm^2^ data through the embedded immediate post-processing software. The DCE-MRI protocol involved injecting gadolinium-diethylenetriamine pentaacetic acid (GD-DTPA, 0.1 mmol/kg) and acquiring images in the five consecutive phases after pre-contrast T1WI. The six phases were named as: pre-contrast (DCE_1_), super-early-contrast (DCE_2_), early-contrast (DCE_3_), and delayed-contrast (DCE_4-6_). The detailed MRI scanning parameters are provided in **Supplementary Materials Table S1**.

### The volume of interest delineation

The volume of interest (VOI) was defined on all images that were stored in DICOM format. In order to standardize the extracted image biomarkers from mpMRI, we followed the major procedure outlined by the Image Biomarker Standardization Initiative (IBSI) (17). Before VOI delineation, we used the General registration (elastix) method, available as the “SlicerElastix” plugin in the open-source image analysis platform 3D Slicer (https://www.slicer.org), to register all sequences’ images. This alignment enabled us to better handle morphological variations and structural differences in breast tissue, particularly when aligning the other sequence images with the DCE_2_ image. Additionally, we resampled all MRI sequences to a standard resolution of 1.096 x 1.096 x 1.2, ensuring isotropic voxels and reducing variations caused by differences in scanning equipment, protocols, and patient positioning. Furthermore, we normalized the intensity levels of all images to a range of 0-255 to reduce the influence of contrast and brightness variations, which might otherwise affect the quantification of radiomics features (18).

Slice-wise delineation of the VOI was carried out using the ITK-SNAP software (http://www.itksnap.org) on T2W, DWI_600_, DWI_800_, ADC maps, and DCE_1-6_ images. The process started with manual delineation of the visible tumor margins on the DCE_2_ images with the most distinguishable lesion boundary. The contoured VOIs on DCE_2_ were then replicated to the remaining DCE sequences, resulting in 6 VOIs based on the DCE-MRI data. Similar steps were repeated for the DWI_600_, DWI_800_, and ADC maps. Subsequently, VOI delineation was performed on the T2W images according to the position and shape of the VOI completed above. Slight adjustments were allowed on all images in order to obtain tailored VOIs. The VOI delineation was performed by two radiologists (WZ and JL, with 6 and 4 years of experience in radiological diagnosis, respectively) who were blind to all prior patient information. The interobserver correlation coefficient (ICC) value of the two radiologists was assessed.

### Radiomics feature extraction and analysis

The radiomics features were extracted from ten VOIs of each lesion using the open-source software toolkit Pyradiomics (19). A total of 109 features were extracted from three categories of features: 1) intensity features (n=19); 2) morphology features (n=15); texture features (n=75). Only the extracted radiomics features with ICC > 0.75 were then fed into 150 classification base models, which were built using 10 classifiers and 15 feature selection methods. Detailed definitions of the above-mentioned features can be found in Pyradiomics documentation and IBSI (17). The full list of radiomics features and the methods employed in this study are summarized in **Tables S2**, **S3**, respectively.

### Feature fusion radiomics modeling and evaluation

Based on the newly developed mpMRI-based RadioFusionOmics model by our lab, we constructed a feature fusion radiomics (R_FF_) model that integrated radiomics information from different MRI sequences to produce more discriminative fused features. A random selected training cohort (n = 337) was used to analyze all radiomics features from each MRI sequence, analogous to a radiologist’s initial reviewing of a patient’s complete set of MR images. According to the highest cross-validation AUC obtained in the training/validation process, the optimal single sequences that can identify hormone receptor positive (HR+) *vs*. HR- BC, TNBC *vs*. HEBC, as well as TNBC *vs*. non-TNBC were determined and regarded as the single sequence-based radiomics (Rss) model.

**Figure 2:**
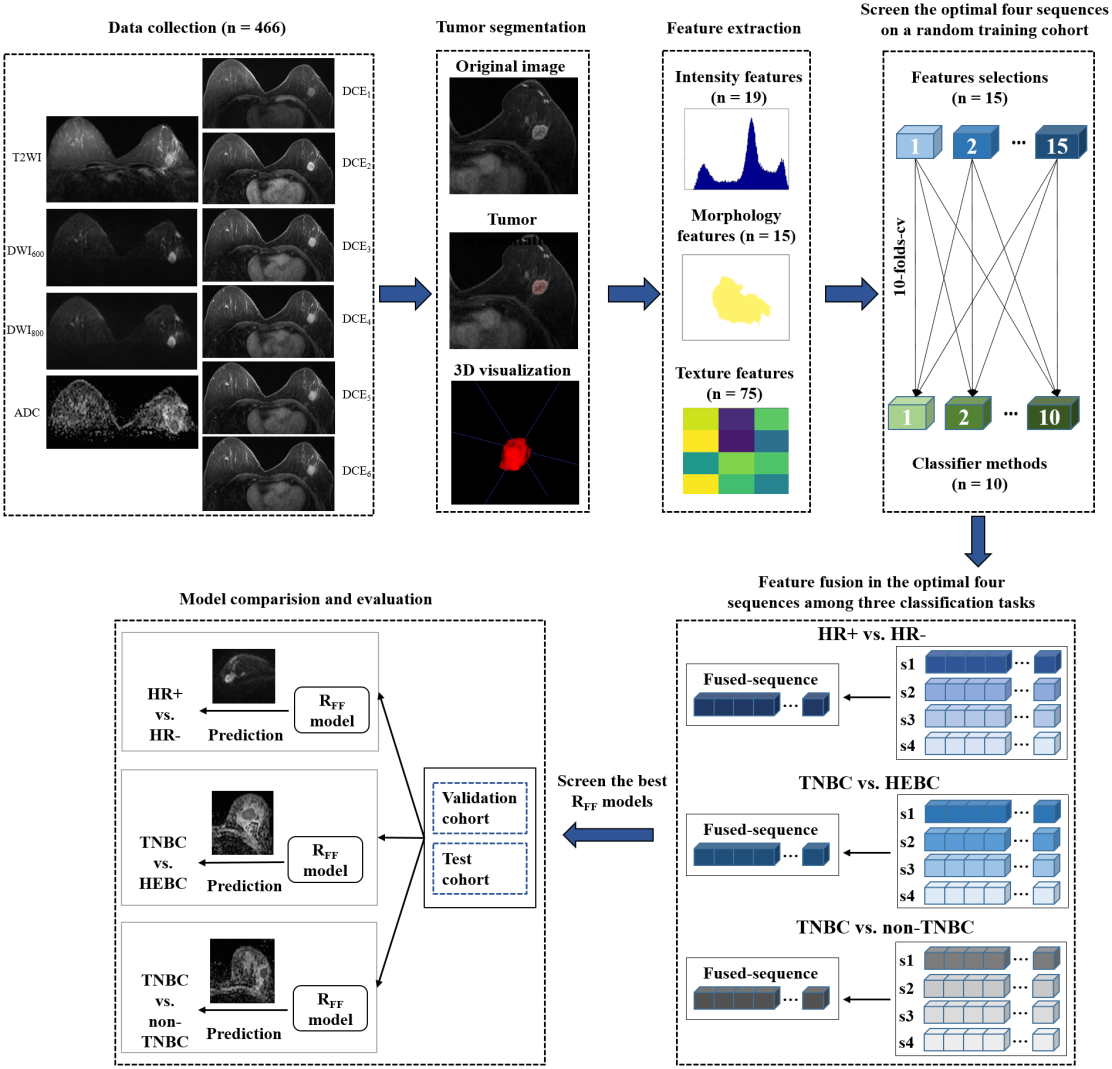
Flow chart of the study. HR, hormone receptor; TNBC, triple-negative breast cancer; HEBC, human epidermal growth factor receptor 2 enriched BC.

Subsequently, the radiomics features from the top four high-performing single sequences were combined to perform multiple sequence feature fusion, similar to a radiologist’s final reviewing focusing on specific sequences after a preliminary review. The best combination of sequences (combination of two, three or four sequences, a total of 11 types of combinations) was then identified to develop the R_FF_ models. Utilizing feature-level fusion, the R_FF_ model conducted a feature-wise fusion strategy by finding a transformation to map the feature matrix with a set of MRI sequences (e.g., dimension = 10) to a lower dimensional space (e.g., dimension = 1). By integrating the class structure information (i.e., information on the molecular receptor status of memberships of the training samples) in the calculation of the transformation, the R_FF_ was able to eliminate the between-class correlations and strengthen the within-class correlations during the feature fusion, which can effectively enhance the discriminative power of fused features. Various base models (n=11*150 = 1650) were trained using the fused features and their performances were evaluated and ranked via a stratified ten-fold cross-validation. The optimal base models for Rss and R_FF_ were verified in the training/validation cohort and test cohort. Technical details related to the R_FF_ are shown in **Appendix 2**. The flow chart of this study was displayed in **Figure 2**.

### Histopathology

All surgical or biopsy specimens were examined by two pathologists (YZ and WD, with 6 and 16 years of experience in the pathological diagnosis of BC, respectively). The following pathological biological markers of BCs were assessed and recorded: tumor maximal diameter, affected side in the breast, number of tumors, histology type, and IHC status of estrogen receptor (ER), progesterone receptor (PR), HER-2, and Ki-67 index. Tumors with ER or PR positive expression (> 10% of tumor nuclei staining) were classified as HR positive (HR+) (20). Positive HER-2 expression was defined as a 3+ IHC score or 2+ accompanied by fluorescence *in situ* hybridization positive (FISH+) result (21). The Ki-67 scores were classified into two groups: < 14% as low Ki-67 level and ≥14% as high Ki-67 level. The molecular subtypes of BCs were classified as follows: luminal A (ER and/or PR positive, HER-2 negative, and Ki-67 < 14%), luminal B (ER and/or PR positive, HER-2 negative, and Ki-67 ≥ 14% or ER and/or PR positive, HER-2 positive, regardless Ki-67 expression), HER-2 enriched (ER and PR negative, HER-2 positive), which was recorded as HEBC, and triple negative cancer (ER, PR and HER-2 negative), named as TNBC. The luminal A and luminal B comprised the HR+ group. The Ki-67 expression was scored as the percentage of positive invasive tumor cells with any nuclear staining, with the mean percentage of positive cells recorded (4). Four cases of different molecular subtypes of breast cancer were presented in the **supplementary materials Figures S3**-**S6**.

### Statistical analysis

The Chi-square Test and Fisher’s Exact Test were used for categorical variables, the One-way ANOVA analysis was used for normally distributed continuous variables, and the Kruskal-Wallis H test was used for non-normally distributed continuous variables to compare demographic and pathological characteristics between different molecular subtypes. The normality of data distribution was evaluated by the Shapiro-Wilk test. The results for normally distributed continuous variables were reported as mean ± SD, while non-normally distributed continuous variables were reported as median (interquartile range, IQR). Categorical variables were presented as numbers and proportions. The performance of each Rss and R_FF_ base models were evaluated via the area under the receiver operative characteristic curve (AUC), sensitivity (SEN), specificity (SPE) and accuracy (ACC) among different subtypes of BC. The performance of the Rss and R_FF_ was compared using the paired samples Wilcoxon signed rank test. Two-sided *p* < 0.05 was considered statistically significant. All statistical analyses were conducted using the SPSS 25.0 software (IBM SPSS Corporation, USA) and python 3.6.2 (Python Software Foundation (USA, https://www.python.org/downloads/).

## Results

### Demographics data and tumor characteristics

The clinical pathological characteristics of the 460 patients with 466 lesions (6 patients had bilateral lesions) enrolled in the study are presented in **Table 1**. Among the 466 lesions, 336 lesions (72.1%) were classified as HR+ BCs, with 142 lesions being luminal A and 194 lesions being luminal B. Additionally, 76 lesions (16.3%) were classified as HEBCs, and 54 lesions (11.6%) were classified as TNBCs. The median tumor size of TNBCs (26.0 mm) and HEBC (27.0 mm) was found to be significantly larger than that of HR+ (21.0 mm) (*p* = 0.000). TNBCs showed a higher prevalence of mass enhancement in DCE MRI (81.5%) and invasive carcinoma (96.2%) compared to HR+ and HEBCs (*p* < 0.001). TNBCs also had a higher Ki-67 index (> 14%) in comparison with HR+ and HEBCs. Moreover, the age of patients and number of tumors among HR+, HEBC and TNBC groups were significantly different (*p* < 0.05). Baseline characteristics were not significantly different between both training/validation and test cohorts (**Table S4**).

**Table 1:** Demographics data and tumor characteristics.

characteristics	HR+ (n=336)	HEBC (76)	TNBC (n=54)	*P* values
age	55.77 ± 11.00	54.58 ± 9.73	51.43 ± 12.09	**0.024** ^a^
tumor size (mm)*	21.00 (16.00-30.00)	27.00 (18.25-42.75)	26.00 (19.75-39.25)	**0.000** ^b^
affected side				0.119^c^
left	167 (49.7)	47 (61.8)	25 (46.3)	
right	169 (50.3)	29 (38.2)	29 (53.7)	
tumor enhancement morphology				**0.004** ^c^
mass enhancement	317 (94.3)	69 (90.8)	44 (81.5)	
non-mass enhancement	19 (5.7)	7 (9.2)	10 (18.5)	
number of tumor				**0.001** ^c^
single	233 (69.3)	37 (48.7)	40 (74.1)	
multicentric or multifocal	103 (30.7)	39 (51.3)	14 (25.9)	
Ki-67 status				**0.000** ^c^
<14%	123 (36.6)	5 (6.6)	1 (1.9)	
>14%	213 (63.4)	71 (93.4)	53 (98.1)	
histological type				**0.000** ^d^
invasive carcinoma	294 (87.5)	62 (81.6)	52 (96.2)	
ductal carcinoma in situ	22 (6.6)	14 (18.4)	1 (1.9)	
intraductal papillary lesions	18 (5.5)	0 (0)	0 (0)	
others†	1 (0.4)	0 (0)	1 (1.9)	

Unless indicated otherwise, data are numbers of cancers, with percentages in parentheses.

*Data are median, with interquartile range (IQR) in parentheses.

†Other invasive cancers are 1 neuroendocrine carcinoma in HR+ and 1 malignant phyllodes tumor carcinoma in TNBC.

a One-way ANOVA analysis.

b Kruskal-Wallis H test.

c Chi-square test.

d Fisher’s Exact Test.

A P value less than 0.05 was considered statistically significant, presented in **bold**. HR hormone receptor, HEBC human epidermal growth factor receptor 2 enriched breast cancer, TNBC triple negative breast cancer.

### Selection of the dominant sequence and development of the Rss model

All the discriminative base models established based on single mpMRI sequence were compared to determine the optimal sequences among HR+ *vs*. HR-, TNBC *vs*. HEBC and TNBC *vs*. non-TNBC. **Supplementary Figure S1** demonstrated the discrimination comparison results on ten sequences of the three classification tasks.

**Figure 3:**
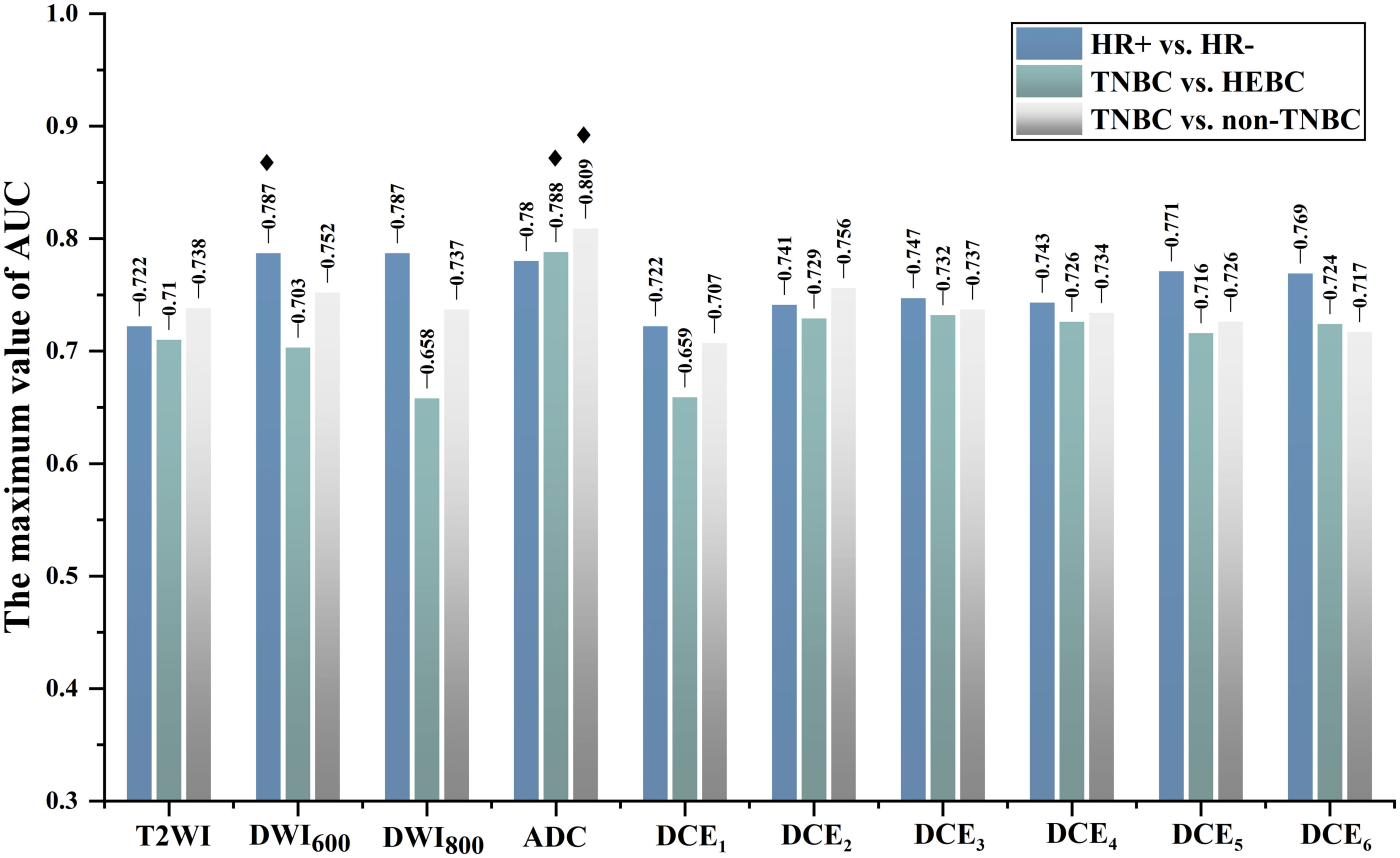
Each sequence with the maximum AUC value in identifying HR+ *vs*. HR- (DWI_600_), TNBC *vs*. HEBC (ADC), and TNBC *vs*. non-TNBC (ADC) in the random training cohort. HEBC, human epidermal growth factor receptor 2 enriched BC;TNBC, triple-negative breast cancer; HR, hormone receptor. ◆ The optimal single sequence for each classification.

By analyzing the dominant radiomics features of each sequence, the optimal sequence for discriminating HR+ *vs*. HR- was DWI_600,_ the optimal Rss model, namely Rss (DWI_600_), achieved the highest AUC of 0.787 in the random training cohort (**Figure 3**), and similar performance in the training/validation cohort (AUC=0.767) and test cohort (AUC=0.768), respectively (**Table 2**).

**Table 2:** performance of the optimal Rss model and the optimal R_FF_ model for different molecular receptor statuses discrimination.

Classification tasks	Model	Training/Validation cohort					Test cohort				
		AUC	ACC	SEN	SPE	*P* value	AUC	ACC	SEN	SPE	*P* value
HR+ *vs*. HR-	Rss (DWI_600)_	0.767	0.736	0.770	0.653		0.768	0.693	0.689	0.706	
	R_FF_ (DWI_600_+DWI_800_+DCE_5_)	0.778	0.737	0.753	0.699	0.066	0.726	0.659	0.673	0.613	**0.028**
TNBC *vs*. HEBC	Rss (ADC)	0.769	0.656	0.667	0.650		0.718	0.692	0.684	0.700	
	R_FF_(ADC+DCE_2_+DCE_4_)	0.787	0.692	0.655	0.720	**0.043**	0.773	0.645	0.636	0.650	**0.017**
TNBC *vs*. non-TNBC	Rss (ADC)	0.784	0.727	0.683	0.734		0.735	0.707	0.611	0.721	
	R_FF_(ADC+DWI_600_+T2WI+DCE_2_)	0.818	0.718	0.705	0.721	**0.042**	0.773	0.767	0.636	0.780	**0.025**

P value: compared the performance between the optimal Rss model and the optimal R_FF_ model in the training/validation cohort and test cohort of each discriminative task. Significant values (P < 0.05) are presented in **bold**. HR, hormone receptor; HEBC: human epidermal growth factor receptor 2 enriched BC; TNBC: triple-negative breast cancer. AUC, area under the receiver-operating characteristic curve; SEN, sensitivity; SPE, specificity; ACC, accuracy.

The optimal sequence for identifying TNBC and HEBC was DWI-derived ADC map, the best Rss model, recorded as Rss (ADC), yield the highest AUC of 0.788 in the random training cohort (**Figure 3**), and the best AUC of 0.769 and 0.718 in the training/validation cohort and test cohort, respectively (**Table 2**).

Regarding TNBC *vs*. non-TNBC discrimination, the ADC map was also the best sequence, the optimal Rss model (Rss [ADC]) demonstrated the highest AUC of 0.809 in the random training cohort (**Figure 3**), and the best AUC of 0.784 and 0.735 in the training/validation cohort and test cohort, respectively (**Table 2**).

### R_FF_ model development and evaluation

We selected the top four superior sequences for molecular receptor status classification to build the R_FF_ model. As shown in **Figure 3** and **Figure S1**, the top four superior sequences for HR+ *vs*. HR- were DWI_600_, DWI_800_, DWI-derived ADC map and DCE_5,_ with all AUCs > 0.77 in the random training cohort (**Figure S1A**). Similarly, DWI-derived ADC map, DCE_2_, DCE_3_ and DCE_4_ were the top four dominant sequences for TNBC *vs*. HEBC, yielding all AUCs > 0.72 (**Figure S1B**). While the four most predominant sequences for TNBC *vs*. non-TNBC were DWI-derived ADC map, DWI_600_, T2WI and DCE_2_, achieving all AUCs greater than 0.73 (**Figure S1C**).

Subsequently, the performances of each combination of the top two, three or four high-performance mpMRI sequences in random training cohort (a total of 11 types of combinations during each classification task) were compared and displayed in **Figure S2**. Our results illustrated that the model R_FF_ (DWI_600_+DWI_800_+DCE_5_), R_FF_ (ADC+DCE_2_+DCE_4_) and R_FF_ (ADC+DWI_600_+T2WI+DCE_2_) were superior over the other sequences combinations in the random training cohort, yielding the maximal AUC of 0.809, 0.805 and 0.847, respectively. Similar performances were obtained in the training/validation cohort and test cohort, outperforming the Rss model with an AUC of 0.778 and 0.726, 0.787 and 0.773, 0.818 and 0.773, respectively (both *p*<0.05 except HR+ *vs*. HR-), as shown in **Table 2**. Among R_FF_ (DWI_600_+DWI_800_+DCE_5_), R_FF_ (ADC+DCE_2_+DCE_4_) and R_FF_ (ADC+DWI_600_+T2WI+DCE_2_), the base model (classifier + feature selection method) were respectively “Logistic Regression + Multi-Cluster Feature Selection” (MCFS), “Logistic Regression + Discriminative Feature Selection” (UDFS) and “Logistic Regression + trace_ratio”. The MpMRI-based feature fusion method employed in the task of TNBC *vs*. non-TNBC achieved the optimal discriminative capability, yielding AUC, ACC, SEN and SPE of 0.818, 0.718, 0.705, 0.721 in the training/validation cohort and 0.773, 0.767, 0.636, 0.780 in the test cohort, respectively.

### Top-ranked radiomics features

The top-ranked features associated with the three classification tasks were also sieved by the proposed R_FF_ model and their discriminative capabilities were analyzed. Based on the feature selection procedure of each base model, we counted and ranked the occurrence of each selected feature (only for base models with AUC > 0.6). The fifteen most frequently selected features of the three classification tasks were displayed in **Tables S5**-**S7**. Most dominant features were texture features in HR+ *vs*. HR- (8/15) and TNBC *vs*. HEBC (8/15), while intensity-based features were the superior discriminative features of TNBC *vs*. non-TNBC (11/15). The top 5 most frequently selected radiomics features associated with the discrimination of HR+ and HR- included three morphology-based features and two gray level co-occurrence matrix (GLCM) features, while intensity-based features accounted for 80% (4/5) and 100% (5/5), respectively among the top 5 radiomics features of TNBC *vs*. HEBC and TNBC *vs*. non-TNBC (**Table 3**). All the features showed statistically significant differences between HR+ and HR-, TNBC and HEBC, TNBC and non-TNBC with *p*-values < 0.001. The mean feature values of each group were used as the threshold to identify different molecular receptor statuses. In the task of discriminating TNBC from non-TNBC, the top 5 features outperformed other two tasks, with ~75% of the non-TNBC having larger feature values, while ~65% of the TNBC group had smaller values in all top 5 features (**Table 3**).

**Table 3:** The top 5 most frequently selected radiomics features of the three classification tasks based on the optimum R_FF_ models.

Classification tasks	Top 5 radiomics features	*P* value	M	(<Mean |>Mean)
HR+ *vs*. HR-	shape_SphericalDisproportion (1st)	0.0002	0.569	HR+ (70.59% | 29.41%)
				HR- (57.58% | 42.42%)
	shape_MinorAxisLength (2nd)	0.0013	0.203	HR+ (64.29% | 35.71%)
				HR- (49.49% | 50.51%)
	shape_Sphericity (3rd)	0.0002	-0.691	HR+ (72.27% | 27.73%)
				HR- (56.57% | 43.43%)
	glcm_Correlation (4th)	0.0002	0.729	HR+ (57.56% | 42.44%)
				HR- (39.39% | 60.61%)
	glcm_Imc2 (5th)	0.0010	1.069	HR+ (57.14% | 42.86%)
				HR- (41.41% | 58.59%)
TNBC *vs*. HEBC	firstorder_Entropy (1st)	<10^-4^	-1.229	**TNBC (67.44% | 32.56%)**
				**HEBC (30.36% | 69.64%)**
	firstorder_MeanAbsoluteDeviation (2nd)	0.0004	-0.778	TNBC (55.81% | 44.19%)
				HEBC (30.36% | 69.64%)
	firstorder_Uniformity (3rd)	<10^-4^	0.838	**TNBC (69.77% | 30.23%)**
				**HEBC (35.71% | 64.29%)**
	glcm_ClusterTendency (4th)	0.0017	-0.390	TNBC (58.14% | 41.86%)
				HEBC (28.57% | 71.43%)
	firstorder_RobustMeanAbsoluteDeviation (5th)	0.0007	-0.689	TNBC (53.49% | 46.51%)
				HEBC (32.14% | 67.86%)
TNBC *vs*. non-TNBC	firstorder_90Percentile (1st)	<10^-7^	-0.169	**TNBC (62.79% | 37.21%)**
				**non-TNBC (25.17% | 74.83%)**
	firstorder_MeanAbsoluteDeviation (2nd)	<10^-6^	-0.232	**TNBC (62.79% | 37.21%)**
				**non-TNBC (24.83% | 75.17%)**
	firstorder_RobustMeanAbsoluteDeviation (3rd)	<10^-6^	-0.198	**TNBC (62.79% | 37.21%)**
				**non-TNBC (22.45% | 77.55%)**
	firstorder_Entropy (4th)	<10^-6^	-0.365	**TNBC (67.44% | 32.56%)**
				**non-TNBC (30.95% | 69.05%)**
	firstorder_RootMeanSquared (5th)	<10^-5^	-0.103	**TNBC (60.47% |39.53%)**
				**non-TNBC (27.55% | 72.45%)**

The ‘Mean’ shows the mean of the mean radiomics feature values of the two groups in each classification. The letter of ‘(<Mean | >Mean)’ represents the percentage of patients in the two groups with feature value smaller than or larger than the ‘Mean’ value. Values in **bold** indicate these features with better discriminative performance.

## Discussion

Our study aimed to simulate the diagnostic process of radiologists by comprehensively analyzing radiomics features from mpMRI to distinguish different receptor statuses (HR+ *vs*. HR-, TNBC *vs*. HEBC, and TNBC *vs*. non-TNBC) of breast cancers. Initially, the most discriminative MRI sequences (denoted as Rss models) were screened out from the radiomics features, and then the “R_FF_ models” were built by incorporating the top four sequences with high performance on molecular subtype classification. This approach resembles the typical diagnostic process of a radiologist, who first performs a preliminary assessment of all available imaging sequences and then focuses on a subset of sequences with particularly informative features for the final diagnosis. The results showed that the R_FF_ models “DWI_600_+DWI_800_+DCE_5_”, “ADC+DCE_2_+DCE_4_” and “ADC+DWI_600_+T2WI+DCE_2_” outperformed each Rss model in the classification tasks of HR+ *vs*. HR-, TNBC *vs*. HEBC, and TNBC *vs*. non-TNBC, with all AUC values exceeding 0.7. These findings highlight the effectiveness of fusing multi-sequence MRI radiomics features by the R_FF_ approach to achieve high performance in differentiating different receptor statuses of BCs.

Breast cancers exhibit high heterogeneity, leading to distinct therapeutic approaches, such as endocrine therapy for HR+ BCs, targeted therapy with anti-HER-2 monoclonal antibodies for HEBCs, and NST mainly for TNBCs (3). Radiomics, deriving multiple quantitative features from multimodal medical images, may capture spatiotemporal heterogeneity reflected by different molecular receptor statuses before treatment. This improves the discriminative and predictive abilities of medical image in oncology (6, 22). Previous studies have applied radiomics preoperatively to assess molecular receptor statuses of BCs and reported preliminary success (7, 8, 11, 12). For instance, Leithner et al. found that radiomic signatures extracted from DCE-MRI via a K-Nearest Neighbors (KNN) classifier were capable of classifying luminal A *vs*. luminal B, luminal B *vs*. triple negative, luminal B or HER-2 enriched *vs*. all other cancers (all ACC >77%) (11). However, most previous studies employed only one or two MRI sequence(s) such as DCE-MRI or DWI-derived ADC maps, without exploring all routine mpMRI sequences, leading to uncertainty regarding which sequences are more important. Our study compared the performances of all ten routine mpMRI sequences, revealing that radiomics signatures from DWI_600_, DWI_800_, DWI-derived ADC map, and DCE_5_ sequences exhibited superior discriminative power for HR+ *vs*. HR-, especially the DWI_600_ and DWI_800_ sequences. Interestingly, radiomics features from DWI-derived ADC maps contributed more than other sequences for TNBC *vs*. HEBC and TNBC *vs*. non-TNBC.

The DWI provides a quantitative ADC parameter that closely reflects the microenvironment of tumor structures such as tumor cellularity, fluid viscosity, the amount of fibrous stroma, and cell membrane permeability, by detecting the Brownian motion of water molecules (23, 24). DWI and ADC maps have been widely used in tumor characterization, particularly in BC. While previous studies have conducted quantitative analyses based on ADC maps to identify different molecular receptor statuses or subtypes of BC, however, the reported results were inconsistent (25–29). For example, Suo et al. found that HER-2 positive subtype exhibited higher mean ADC values than other subtypes of BC with either standard (800 s/mm^2^) or high (1500 s/mm^2^) b-values (26). However, other studies have reported that TNBC had a higher mean ADC value than other subtypes (28, 29). These inconsistent findings may be due to the use of different b-values in DWI, different ROI selection strategies (e.g., 2D or 3D ROIs, ROI containing the whole tumor or the lower part of ADC values within the lesion), variations in magnetic field, etc. (27, 30, 31). Further studies and investigations are warranted, but these trends in ADC values according to clinically relevant subtypes may provide potential imaging biomarkers to aid treatment decisions in BC in the future. The results of our comprehensive analysis revealed that ADC map and DWI sequences played a dominant role in the three classification tasks, suggesting that radiologists should give greater attention to ADC maps and DWI sequences during the clinical interpretation process.

In addition, we found that the DCE_5_ sequence, one of the delayed-contrast phases, was more important than other DCE phases in the differentiation between HR+ and HR- BCs. Generally, a time-signal intensity curve on DCE-MRI with a rapid enhancement (corresponding to DCE_2-3_ in our study) followed by a washout pattern, is generally indicative of a malignant breast lesion. However, this pattern does not apply to TNBC, which is a common HR- subtype. A previous study showed persistent enhancement pattern on DCE-MRI was significantly associated with TNBC (32). Interestingly, another research showed that a significant proportion (33% [25 of 76]) of familial BCs exhibited a slow or intermediate initial enhancement followed by steady delayed enhancement pattern, which was the general DCE-MRI kinetic feature for benign BC lesions (33). This discrepancy in DCE-MRI enhancement patterns between HR+ and HR- subtypes may be explained by their unique pathohistological features (34, 35). ER-negative BCs are known to have several unique histological features, such as prominent lymphoid stroma, comedo-type necrosis, and central fibrosis (34). TNBC is also highly associated with the presence of a central scar, tumor necrosis, and stromal lymphocytic response (35). These features may result in retaining of contrast agent within the center of lesions and show persistent enhancement, which may be captured as dominant radiomics features from the delayed-contrast phase of DCE-MRI. Our results suggested the potential of the delayed-contrast phase of DCE-MRI in differentiating HR+ and HR- subtypes and in the selection of endocrine therapy candidates.

In this study, we explored the potential of fusing dominant features from mpMRI sequences to improve the accuracy of BC subtype classification. Our hypothesis was that multi-dimensional image information from multiple MRI sequences could be captured and integrated to provide a more comprehensive representation of the breast lesion. Different from previous studies (7, 14, 36), we investigated all sequences of a routine breast MRI examination and selected the top four high-performance sequences to develop a discriminative model via fusing dominant features of multi-sequences. Incorporating class structure information, the R_FF_ can not only effectively integrate features from different MR sequences, but also ensures that the fused features are more representative and discriminative. The results of our study emphasized the importance of incorporating multiple MRI sequences in the radiomics analysis of breast cancer, as it can lead to improved accuracy in molecular subtype classification.

Our results showed that the top 5 radiomics features that effectively differentiated HR+ and HR- BC were three morphology-based features and two GLCM-based features. This aligns with prior studies, which have shown that molecular subtypes of BC exhibit distinct morphological and textural characteristics on MRI images (11, 37). Tumors of the luminal type, for instance, tend to present with irregular shapes and irregular/spiculated contours on MRI due to their slow growth rate and the desmoplastic reaction of the surrounding tissue (10, 33). On the contrary, rapidly growing TNBCs and HEBCs tend to have well-defined, oval/round shapes with smooth outlines (10, 32). According to IBSI, GLCM represents the distribution of intensities of neighboring pixels along image directions and reflects the heterogeneity of image intensity (17). Previous studies have shown that BC subtypes also exhibited distinct ADC values, DWI manifestations and enhancing intensity patterns (11, 27, 38–40). A recent study also reported that non-TNBCs had significantly higher mean/median/5^th^ percentile washin values compared to TNBCs, indicating that HR+ and HR- lesions have different intensity-derived radiomics features (41). Of note, first order features accounted for 80% (4/5) and 100% (5/5) for classifying TNBC *vs*. HEBC and TNBC *vs*. non-TNBC, respectively. The intensity statistical features described intensity distribution within the ROI and also reflected tumor’s heterogeneity (11, 42).

## Limitations

Our study has certain inherent limitations that merit acknowledgment. First, the retrospective design and single-center setting of this study was subjected to selection bias. Conducting a multi-center study was not feasible due to the variations in MRI scan protocols across medical centers, necessitating the inclusion of DCE-MRI with 6 different phases and DWI with b values of 600 and 800 mm^2^/s. Second, a majority of tumors with non-mass enhancement in DCE-MRI were excluded due to challenges in defining the boundaries for VOI delineation, potentially introducing further selection bias. Third, the manual delineation of tumors in this study is time-consuming and prone to subjectivity, and future studies will incorporate semi- or automatic segmentation techniques to enhance objectivity. Fourth, not all radiomics features were analyzed, e.g., gray level dependence matrix (GLDM) being beyond the scope of IBSI was excluded. Fifth, we included a subset of breast cancers that were pathologically confirmed through needle biopsy, which may introduce inherent bias of needle biopsy. Finally, the biological interpretability of the “fused features” used in R_FF_ model was insufficient as a result of implementing the feature fusion strategy, which we will focus on in our future studies.

## Conclusion

In conclusion, the R_FF_ model was successfully developed by integrating mpMRI image information to determine different molecular receptors of breast cancer preoperatively. This model, which mimics the diagnostic work pattern of radiologists, outperformed single MR sequence-based radiomics models to distinct molecular receptor status.

## Data availability statement

The original contributions presented in the study are included in the article/**Supplementary Material**. Further inquiries can be directed to the corresponding authors.

## Ethics statement

This study was approved by the Ethics Committee of the Second Affiliated Hospital of South China University of Technology (Guangzhou First People’s Hospital) with informed consent being waived due to the retrospective nature of this study. The studies were conducted in accordance with the local legislation and institutional requirements. The ethics committee/institutional review board waived the requirement of written informed consent for participation from the participants or the participants’ legal guardians/next of kin due to the retrospective nature of this study.

## Author contributions

SL, FL, and WZ contributed equally to this work and shared first authorship. XZ and RY contributed equally and shared correspondence authorship. Literature search: SL, FL, WZ, JZ, and RY. Study design: SL, FL, WZ, RY and XZ. Data collection: SL, FL, WZ, JL, WD, YuZ, and YX. Data analysis: WZ, RY, XZ, FL, SL, WD, JZ, YaZ, and YX. Data verification: all authors. Manuscript editing: SL, FL, WZ, RY and XZ. Manuscript review: All authors. All authors contributed to the article and approved the submitted version.
