# Supplementary material for: Evaluation of molecular receptors status in breast cancer using an mpMRI-based feature fusion radiomics model: mimicking radiologists’ diagnosis
Source: Front Oncol. 2023 Nov 21;13:1219071. doi: 10.3389/fonc.2023.1219071 (PMC10698551; doi:10.3389/fonc.2023.1219071)
Supplement: Supplementary file 1 [file DataSheet_1.docx]

Supplementary Material

# Supplementary Data

**Appendix 1. Details of the sample size calculation**

G*Power (version 3.1.9.6) was used to examine the sample size of the training set. The results showed that the sample size of the training set was sufficient to detect statistical differences among various molecular subtypes of breast cancer. Existing literature suggests that the prevalence of TNBC ranges from approximately 10% to 20%, representing the lowest incidence within the spectrum of breast cancer. Therefore, based on the incidence of TNBC and using an independent two-sample t-test at a significance level of 0.05 and a Cohen's d effect size of 0.5, the sample allocation was determined to be 1:4, resulting in a required sample size of 210 (comprising 42 TNBC cases and 168 non-TNBC cases). Thus, a sample size of 210 is sufficient to detect differences between the TNBC and non-TNBC groups with a statistical power of 95%.

Furthermore, it has been observed that the prevalence of hormone receptor positive (HR+) is approximately 60% to 70%. Consequently, a two-sample t-test with a significance level of 0.05 and a Cohen's d effect size of 0.5 was employed to investigate potential disparities between HR+ and HR- groups. To ensure adequate statistical power, the sample allocation was set at 7:3, leading to a required sample size of 210 (consisting of 147 HR+ cases and 63 HR- cases). Therefore, a sample size of 210 is deemed sufficient to achieve a power of 95% for detecting differences between the HR+ and HR- groups.

Similarly, based on the reported prevalence rates of TNBC (10% - 20%) and HER2-enriched breast cancer (HEBC) (15% - 25%) among breast cancer, utilizing a significance level of 0.05 and a Cohen's d effect size of 0.5, the sample allocation was determined to be 1:1:4:4 (corresponding to TNBC: HEBC: Luminal A: Luminal B), generating a total required sample size of 210 cases, with 21 cases each for TNBC and HEBC. Consequently, a sample size of 42 is deemed sufficient to detect statistically significant differences between TNBC and HEBC, with a power of 95%.

In our study, a total of 238 lesions of HR+, 56 lesions of HEBC, and 43 lesions of TNBC were enrolled. Thus, the sample size of this investigation was sufficient and reasonable. Furthermore, we have provided comprehensive information regarding the calculation of the sample size in both the revised manuscript (page 4, lines 122-125) and supplementary materials (Appendix 1).

**Appendix 2. Details of the feature fusion radiomics (R_FF_) model**

In this study, a total of 109 radiomics features were extracted from each MRI sequence using the Pyradiomics package. For each radiomics feature, a multi-sequence feature matrix $\boldsymbol{X}_{\boldsymbol{p\times n}}$, where $p$ is the number of MRI sequences ($p>2$) and $n$ is the number of training samples, can be constructed. In order to incorporate the class structure information into the fusion process, a between-class scattering matrix $\boldsymbol{S}$ can be constructed by dividing the feature matrix $\boldsymbol{X}_{\boldsymbol{p\times n}}$into $c$ independent groups ($c$ is the number of classes and $c=2$ for this study) according to the various molecular statuses to which the training samples belong. $\boldsymbol{S}$ is calculated as shown in Equation (1).

$S_{p\times p}=\sum_{i=1}^{c} n_{i}\left( \bar{x}_{i}-\bar{x} \right)\left( \bar{x}_{i}-\bar{x} \right)^{T}=\phi\phi^{T}$, (1)

where $n_{i}$is the number of samples belonging to class $i$ in $\boldsymbol{X}_{\boldsymbol{p\times n}} (n=\sum_{i=1}^{c} n_{i})$, ${\bar{\boldsymbol{x}}}_{\boldsymbol{i}}$and$\bar{\boldsymbol{x}}$are the means belonging to class $i$ and all samples in $\boldsymbol{X}_{\boldsymbol{p\times n}}$, respectively. $\boldsymbol{\phi}$ is the covariance matrix, which is calculated in Equation (2).

$\phi=\left[ \sqrt{n_{1}}\left( \bar{x}_{1}-\bar{x} \right), \sqrt{n_{2}}\left( \bar{x}_{2}-\bar{x} \right),\ldots,\sqrt{n_{c}}\left( \bar{x}_{c}-\bar{x} \right) \right].$ (2)

We assume that the$\boldsymbol{S}_{\boldsymbol{c}\boldsymbol{\times}\boldsymbol{c}}^{\boldsymbol{T}}$ (the transpose matrix of $\boldsymbol{S}_{\boldsymbol{p}\boldsymbol{\times}\boldsymbol{p}}$) would be diagonalized if the classes were well-separated. Since the $\boldsymbol{S}_{\boldsymbol{c}\boldsymbol{\times}\boldsymbol{c}}^{\boldsymbol{T}}$ is symmetric positive semidefinite, it can be diagonalized and the eigenvector matrix $\boldsymbol{P}$ and the eigenvalue matrix $\hat{\boldsymbol{\Lambda}}$ are derived as shown in Equation (3).

$P^{T}S^{T}P=\hat{\Lambda}$. (3)

To realize the feature dimensionality reduction ($p\to r$), the eigenvectors corresponding to the first $r$ largest eigenvalues in $\boldsymbol{P}$ are extracted to form the eigenvector $\boldsymbol{Q}$, and the eigenvector matrix $\boldsymbol{\emptyset}\boldsymbol{Q}$ and the eigenvalue matrix $\boldsymbol{\Lambda}_{\boldsymbol{r}\boldsymbol{\times}\boldsymbol{r}}$ of $\boldsymbol{S}$ can finally be obtained by mapping $\boldsymbol{Q}\boldsymbol{\to\emptyset}\boldsymbol{Q}$, as shown in Equation (4).

$\left( \emptyset Q \right)^{T}S\left( \emptyset Q \right)=\Lambda_{\left( r\times r \right)}$. (4)

The unitization of $\boldsymbol{S}$ can generate a transformation matrix $\boldsymbol{W}_{\boldsymbol{p\times r}}$ with the following Equation (5).

$W=\emptyset Q\Lambda^{-1/2}$ (5)

Finally, feature fusion can be accomplished by setting $r=1$ to compress matrix $\boldsymbol{X}_{\boldsymbol{p\times n}}$ to a row vector $\boldsymbol{x}_{\boldsymbol{r\times}\boldsymbol{n}}$ with the transformation of Equation (6).

$x_{r\times n}=W_{r\times p}^{T}X_{p\times n}$. (6)

## Supplementary Figures

**Table S1. MRI scan sequences and parameters**

|  | T2WI | DWI | DCE_1-6_ |
| --- | --- | --- | --- |
| orientation | axial | axial | axial |
| Sequence type | fast spin echo | echo planar imaging | gradient recalled echo |
| TR (ms) | 3800 | 3800 | 5.1 |
| TE (ms) | 42.7 | 78.4 | 2.1 |
| FOV (mm^2^) | 328×350 | 350×200 | 320×320 |
| Matrix | 272×70 | 160×160 | 400×70 |
| Slice thickness (mm) | 4 | 4 | 2.4 |
| Number of slices | 27 | 27 | 120 |
| Interslice gap (mm) | 0.8 | 0.8 | 0.48 |
| number of excitations | 1.6 | 1 | 1 |
| Acquisition time (sec) | 126 | 103 | 394 |

T2WI, T2-weighted imaging. DWI, diffusion-weighted imaging. DCE_1-6,_ six continuous dynamic contrast-enhanced MRI. TR, Time of repetition. TE, time of echo. FOV, field of view.

**Table S2. List of radiomics features**

| **Feature Class** | **Feature Label** |
| --- | --- |
| Intensity (*n*=19) | firstorder_10Percentile, firstorder_90Percentile, firstorder_Energy, firstorder_Entropy, firstorder_InterquartileRange, firstorder_Kurtosis, firstorder_Maximum, firstorder_MeanAbsoluteDeviation, firstorder_Mean, firstorder_Median, firstorder_Minimum, firstorder_Range, firstorder_RobustMeanAbsoluteDeviation, firstorder_RootMeanSquared, firstorder_Skewness, firstorder_TotalEnergy, firstorder_Uniformity, firstorder_Variance, firstorder_StandardDeviation |
| Morphology (*n*=15) | shape_Elongation, shape_Flatness, shape_LeastAxisLength, shape_MajorAxisLength, shape_Maximum2DdiameterColumn, shape_Maximum2DdiameterRow, shape_Maximum2DdiameterSlice, shape_Maximum3Ddiameter, shape_MeshVolume, shape_VoxelVolume, shape_MinorAxisLength, shape_Sphericity, shape_SurfaceArea, shape_SurfaceVolumeRatio, shape_SphericalDisproportion |
| Texture (*n*=75) | glcm_Autocorrelation, glcm_ClusterProminence, glcm_ClusterShade, glcm_ClusterTendency, glcm_Contrast, glcm_Correlation, glcm_DifferenceAverage, glcm_DifferenceEntropy, glcm_DifferenceVariance, glcm_Id, glcm_Idm, glcm_Idmn, glcm_Idn, glcm_Imc1, glcm_Imc2, glcm_InverseVariance, glcm_JointAverage, glcm_JointEnergy, glcm_JointEntropy, glcm_MCC, glcm_MaximumProbability, glcm_SumAverage, glcm_SumEntropy, glcm_SumSquares, glrlm_GrayLevelNonUniformity, glrlm_GrayLevelNonUniformityNormalized, glrlm_GrayLevelVariance, glrlm_HighGrayLevelRunEmphasis, glrlm_LongRunEmphasis, glrlm_LongRunHighGrayLevelEmphasis, glrlm_LongRunLowGrayLevelEmphasis, glrlm_LowGrayLevelRunEmphasis, glrlm_RunEntropy, glrlm_RunLengthNonUniformity, glrlm_RunVariance, glrlm_RunLengthNonUniformityNormalized, glrlm_RunPercentage, glrlm_ShortRunEmphasis, glrlm_ShortRunHighGrayLevelEmphasis, glrlm_ShortRunLowGrayLevelEmphasis, glszm_GrayLevelNonUniformity, glszm_GrayLevelNonUniformityNormalized, glszm_GrayLevelVariance, glszm_HighGrayLevelZoneEmphasis, glszm_LargeAreaEmphasis, glszm_LargeAreaHighGrayLevelEmphasis, glszm_LargeAreaLowGrayLevelEmphasis, glszm_LowGrayLevelZoneEmphasis, glszm_SizeZoneNonUniformity, glszm_SizeZoneNonUniformityNormalized, glszm_SmallAreaEmphasis, glszm_SmallAreaHighGrayLevelEmphasis,glszm_SmallAreaLowGrayLevelEmphasis, glszm_ZoneEntropy, glszm_ZonePercentage, glszm_ZoneVariance, gldm_DependenceEntropy, gldm_DependenceNonUniformity, gldm_DependenceNonUniformityNormalized, gldm_DependenceVariance, gldm_GrayLevelNonUniformity, gldm_GrayLevelVariance, gldm_HighGrayLevelEmphasis, gldm_LargeDependenceEmphasis, gldm_LargeDependenceHighGrayLevelEmphasis, gldm_LargeDependenceLowGrayLevelEmphasis, gldm_LowGrayLevelEmphasis, gldm_SmallDependenceEmphasis, gldm_SmallDependenceHighGrayLevelEmphasis, gldm_SmallDependenceLowGrayLevelEmphasis, ngtdm_Busyness, ngtdm_Coarseness, ngtdm_Complexity, ngtdm_Contrast, ngtdm_Strength |

glcm, gray level co-occurrence matrix. glrlm, gray level run length matrix. glszm, gray level size zone matrix. gldm, gray level dependence matrix. ngtdm, neighbouring gray tone difference matrix. Imc, informational measure of correlation. id, inverse difference. idm, inverse difference moment. idn, inverse difference normalized. idmn, inverse difference moment normalized. MCC, maximal correlation coefficient.

**Table S3. Feature selection methods and classifiers**

| **Feature selection methods(*n*=15)** | Double Input Symmetrical Relevance (DISR), Joint Mutual Information (JMI), fisher_score, reliefF, Spectral Feature Selection (SPEC), trace_ratio, ll_121, ls_l21, Multi-Cluster Feature Selection (MCFS), Nonnegative Discriminative Feature Selection (NDFS), Robust Feature Selection (RFS), Unsupervised Discriminative Feature Selection (UDFS), f_score, gini_index, t_score |
| --- | --- |
| **Classifiers(*n*=10)** | Logistic Regression (LR), K-Nearest Neighbors (KNN), Support Vector Machine (SVM), Gaussian Naive Bayes(GNB), Decision Tree (DT), Extra Trees (ET), Random Forest (RF), Bootstrap Aggregating (Bagging), Adaptive Boosting (AdaBoost), Gradient Boosting Decision Tree (GBDT) |

**Table S4. Clinicopathological characteristics in training/validation and test cohort**

| characteristics | Training/Validation cohort (n=337) | Test cohort (n=129) | *p* |
| --- | --- | --- | --- |
| age | 55.80 ± 11.21 | 56.29 ± 10.07 | 0.141 ^a^ |
| tumor size (mm)* | 20.00 (15.00, 28.50) | 23.00 (18.00, 35.00) | 0.238 ^b^ |
| affected side |  |  | 0.104 ^c^ |
| left | 165 (49.0) | 74 (57.4) |  |
| right | 172 (51.0) | 55 (42.6) |  |
| tumor enhancement morphology |  |  | 0.834 ^c^ |
| mass enhancement | 317 (94.1) | 122 (94.6) |  |
| non-mass enhancement | 20 (5.9) | 7 (5.4) |  |
| number of tumor |  |  | 0.858 ^c^ |
| single | 225 (66.8) | 85 (65.9) |  |
| multicentric or multifocal | 112 (33.2) | 44 (34.1) |  |
| Ki-67 status |  |  | 0.869 ^c^ |
| < 14% | 94 (27.90) | 35 (27.10) |  |
| > 14% | 243 (72.10) | 94 (72.90) |  |
| histological type |  |  | 0.452 ^c^ |
| invasive carcinoma | 286 (84.90) | 113 (87.60) |  |
| non-invasive carcinoma | 51 (15.10) | 16 (12.40) |  |
| molecular subtype |  |  | 0.424 ^d^ |
| HR+ | 238 (70.60) | 98 (76.00) |  |
| HEBC | 56 (16.60) | 20 (15.50) |  |
| TNBC | 43 (12.80) | 11 (8.50) |  |

Unless indicated otherwise, data are numbers of cancers, with percentages in parentheses.

* Data are median, with interquartile range (IQR) in parentheses.

^a^ Student T test

^b^ Mann-Whitney U test

^c^ Chi-square test

^d^ Fisher’s Exact Test

A *P* value less than 0.05 was considered statistically significant, presented in **bold**. HR hormone receptor, HEBC human epidermal growth factor receptor 2 enriched breast cancer, TNBC triple negative breast cancer.

**Table S5. The top fifteen features as calculated by ten classifiers and fifteen feature selection methods of HR+ vs. HR-**

| **Feature Class** | **Feature Label** |
| --- | --- |
| Intensity (*n*=1) | firstorder_Skewness(7th) |
| Morphology (*n*=6) | shape_SphericalDisproportion(1st), shape_MinorAxisLength(2nd), shape_Sphericity(3rd), shape_SurfaceArea(10th), shape_Maximum2DdiameterRow(11th), shape_MajorAxisLength(13th) |
| Texture (*n*=8) | glcm_Correlation(4th), glcm_Imc2(5th), glcm_Autocorrelation(6th), glcm_Idn(8th), glcm_Imc1(9th), glcm_Idmn(12th), gldm_LargeDependenceHighGrayLevelEmphasis(14th), glcm_Contrast(15th) |

**Table S6. The top fifteen features as calculated by ten classifiers and fifteen feature selection methods of TNBC vs. HEBC**

| **Feature Class** | **Feature Label** |
| --- | --- |
| Intensity (*n*=7) | firstorder_Entropy(1st), firstorder_MeanAbsoluteDeviation(2nd), firstorder_Uniformity(3rd), firstorder_RobustMeanAbsoluteDeviation(5th), firstorder_InterquartileRange(6th), firstorder_StandardDeviation(9th), firstorder_10Percentile(14th) |
| Texture (*n*=8) | glcm_ClusterTendency(4th), glcm_SumEntropy(7th), glcm_JointEnergy(8th), glcm_SumSquares(10th), glrlm_GrayLevelNonUniformityNormalized(11th), glcm_JointEntropy(12th), glrlm_HighGrayLevelRunEmphasis(13th), glszm_GrayLevelVariance(15th) |

**Table S7. The top fifteen features as calculated by ten classifiers and fifteen feature selection methods on of TNBC vs. non-TNBC**

| **Feature Class** | **Feature Label** |
| --- | --- |
| Intensity (*n*=11) | firstorder_90Percentile(1st), firstorder_MeanAbsoluteDeviation(2nd), firstorder_RobustMeanAbsoluteDeviation(3rd), firstorder_Entropy(4th), firstorder_RootMeanSquared(5th), firstorder_Energy(6th), firstorder_InterquartileRange(7th), firstorder_Mean(8th), firstorder_Median(9th), firstorder_Uniformity(12th), firstorder_Maximum(14th) |
| Texture (*n*=4) | glcm_SumEntropy(10th),glrlm_GrayLevelNonUniformityNormalized(11th), glszm_HighGrayLevelZoneEmphasis(13th), glrlm_RunLengthNonUniformity(15th) |

c

b


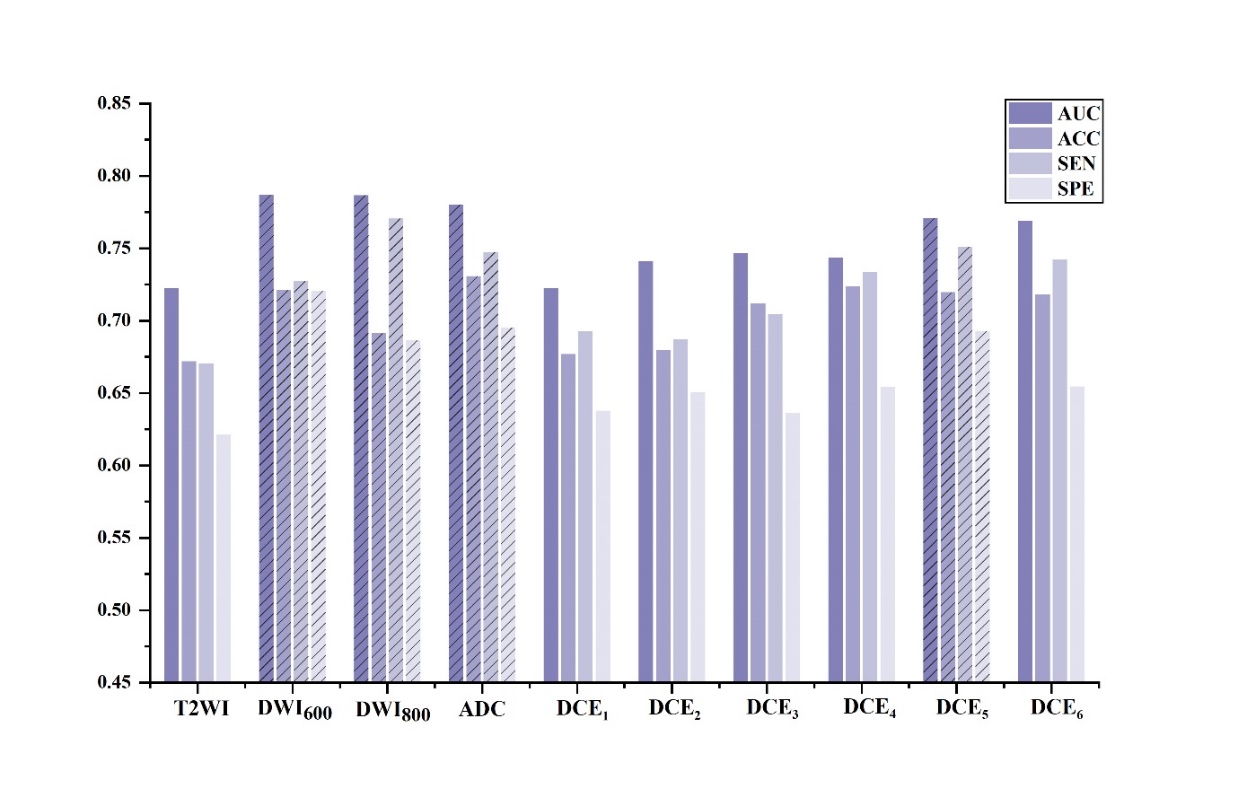

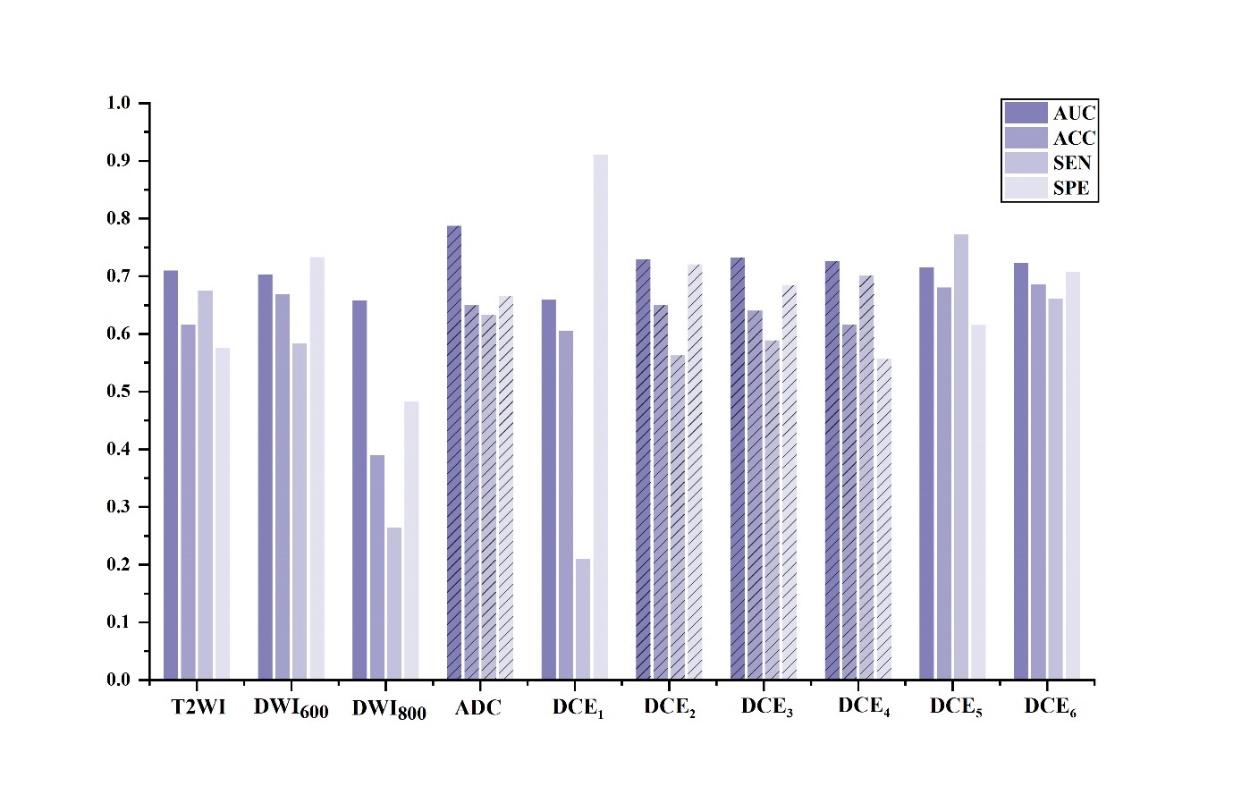

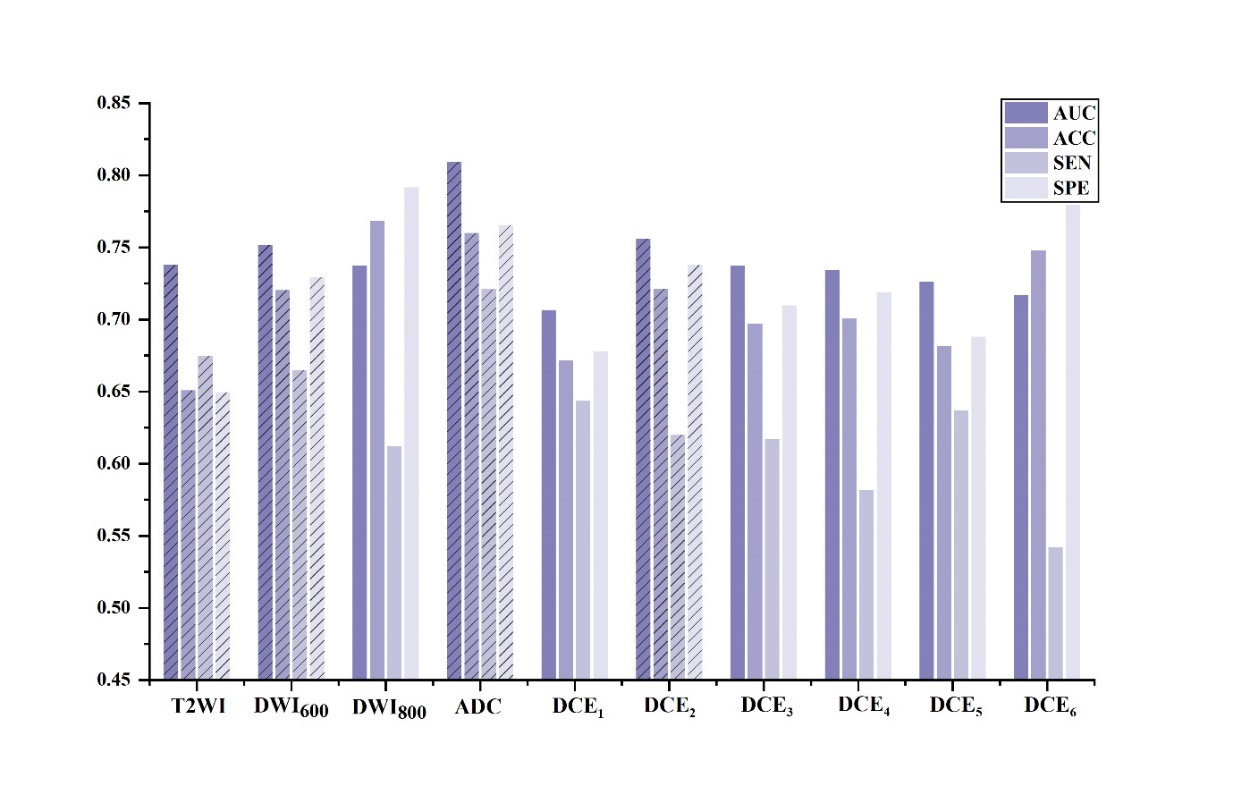


a

Figure S1. The performance of single MRI sequence in the random training cohort to discriminate HR+ vs. HR- (a), TNBC vs. HEBC (b), TNBC vs. non-TNBC (c). The bars filled with diagonal lines represent the four most dominant MRI sequences.





a

b

c







Figure S2. The performance of each combination of the top two, three or four superior sequences compared to the top four single sequence in the random training cohort to discriminate HR+ vs. HR- (a), TNBC vs. HEBC (b), TNBC vs. non-TNBC (c). The bars filled with diagonal lines represent the four most dominant MRI sequences. Asterisks (*) indicate these fused sequences that are statistically different from the optimal single sequence (*P* < 0.05), and the black diamond (◆) indicates the fused sequence with the optimal classification performance.


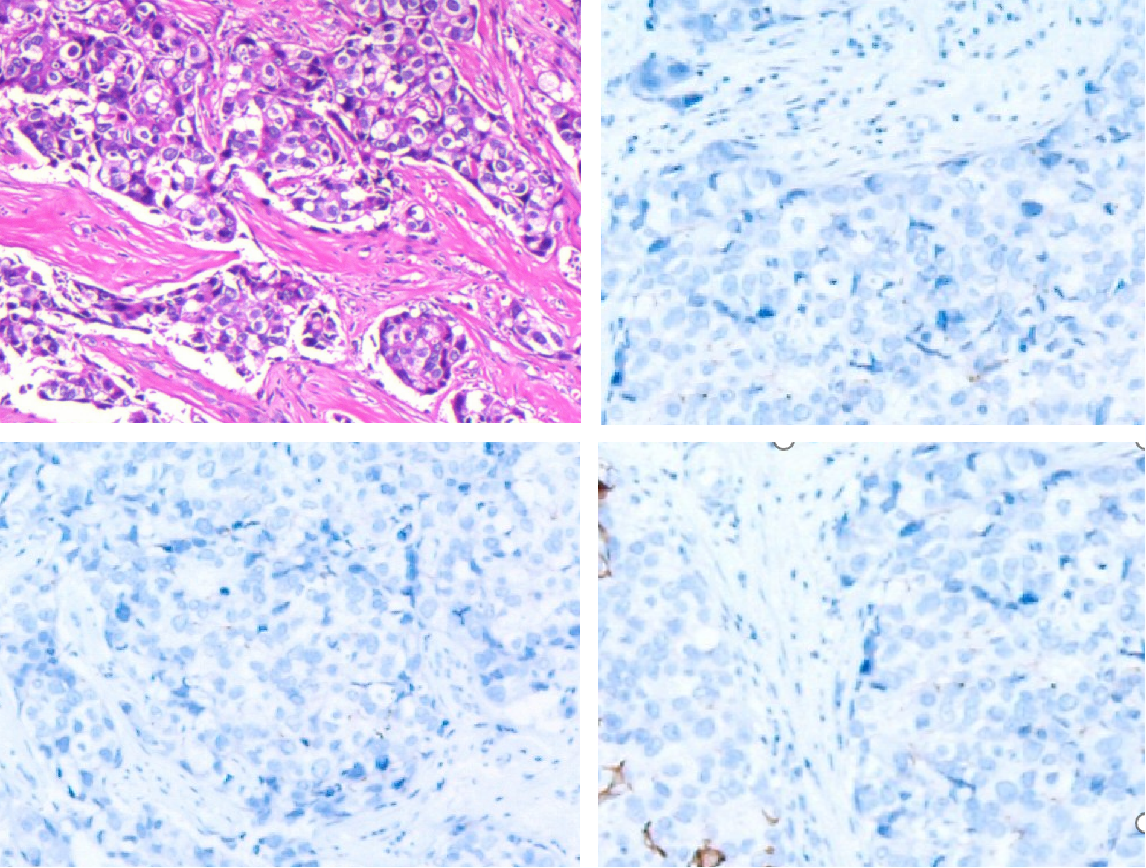


ER

PR

HER-2

HE

Figure S3. A 36-year-old patient with TNBC in her right breast (HE, × 400). Magnification of slides at × 400 showed no expression of ER and PR , along with weak intensity of HER-2 (1+). Ki-67 exhibited 80% of cells strongly positive stained. TNBC, triple-negative breast cancer;ER, estrogen receptor; PR, progesterone receptor; HER-2, human epidermal growth factor receptor 2.


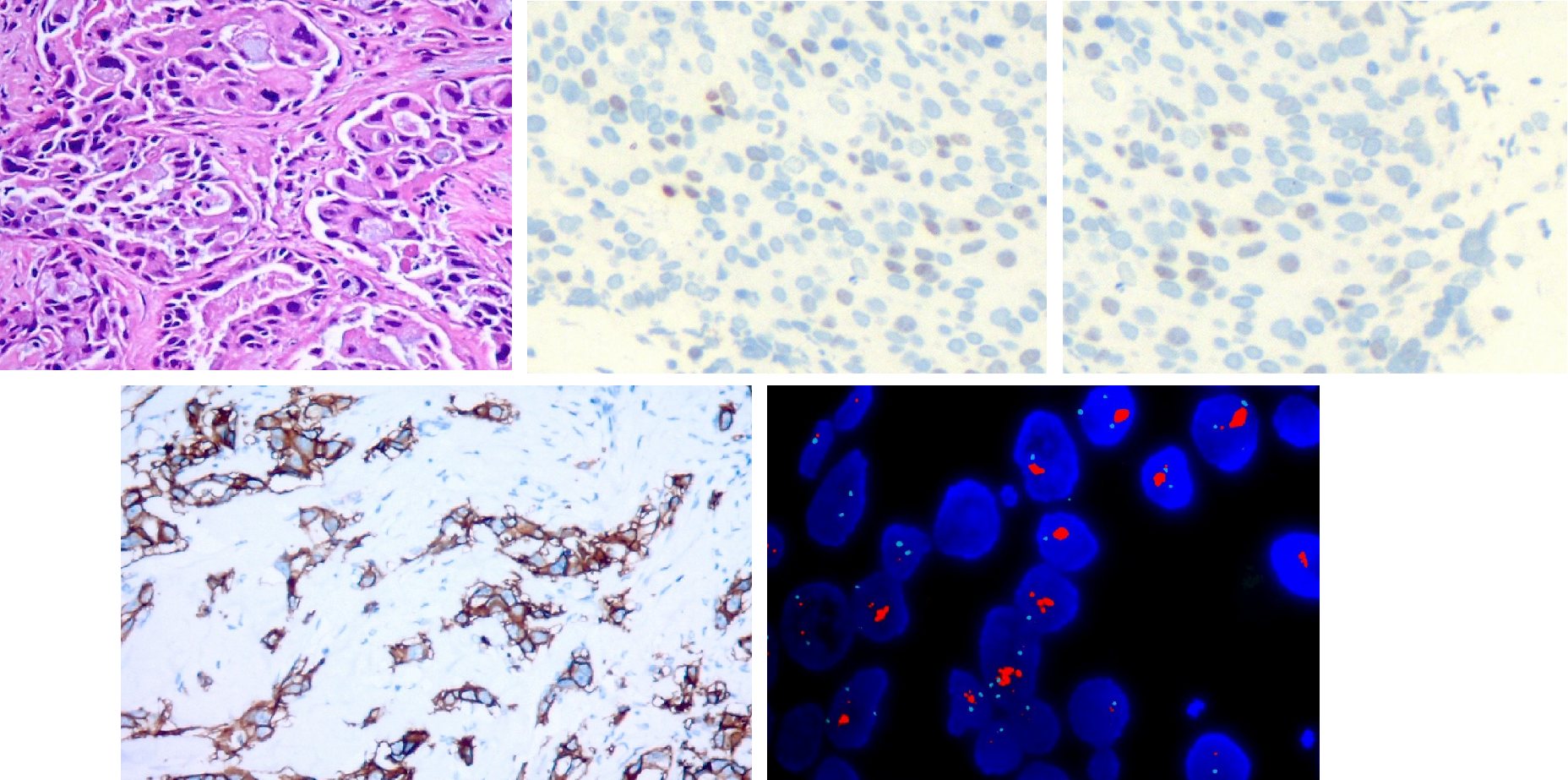


HE

ER

PR

HER-2

FISH

Figure S4. A 54-year-old patient with HEBC in her right breast (HE, × 400). Upon magnification of slides at × 400, the tumor exhibited strong expression of HER-2 (3+) and was confirmed as FISH positive. Week expression of ER (< 10% ) and PR (< 10% ) was found. ER, estrogen receptor; PR, progesterone receptor; HER-2, human epidermal growth factor receptor 2; HEBC; HER-2 enriched breast cancer. FISH, fluorescence in situ hybridization.


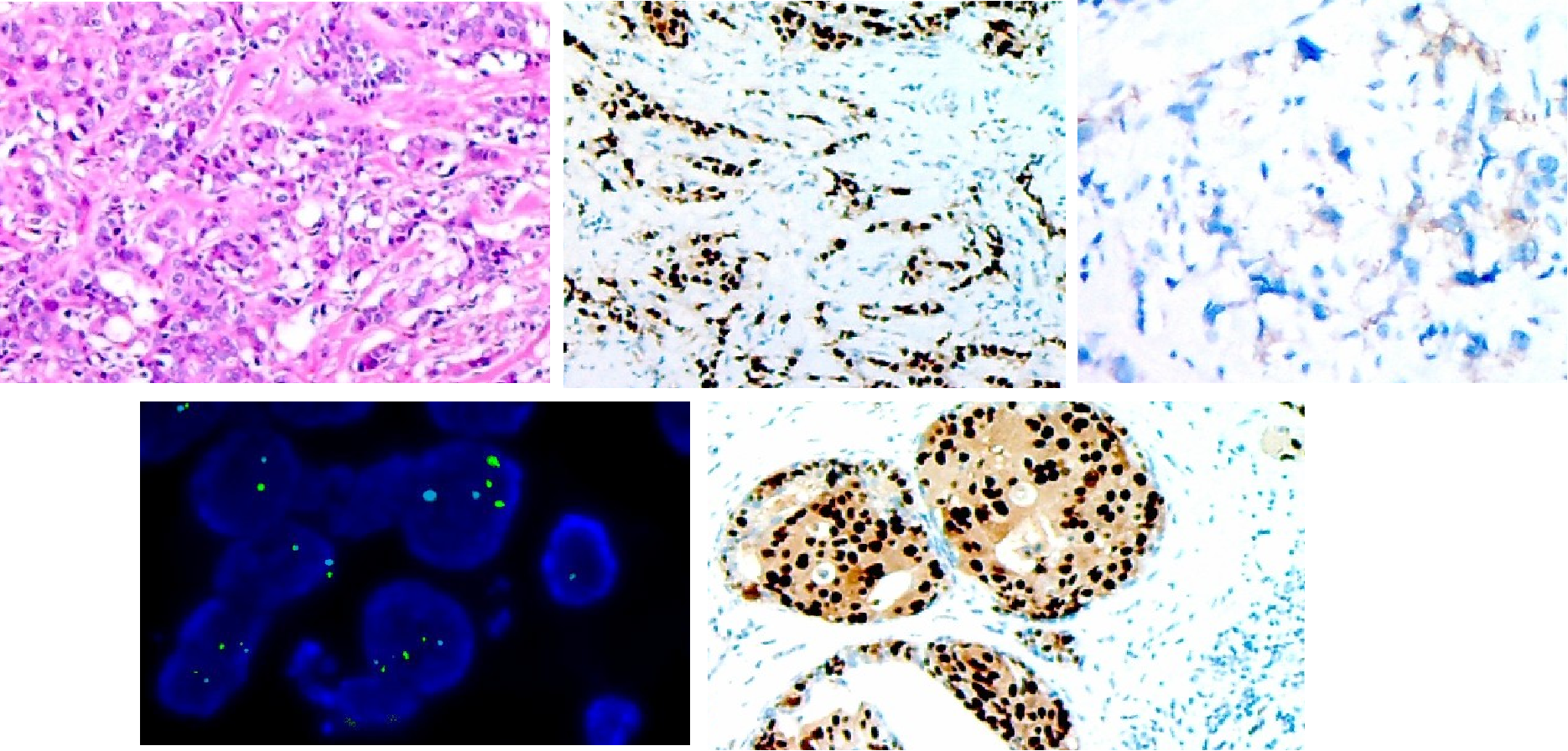


HE

ER

PR

FISH

Ki-67

Figure S5. A 53-year-old patient was diagnosed with luminal B in her right breast (HE, × 200). Strongly expression of ER (~ 60%) and week expression of PR (< 10%) could be observed at a magnification of slides at × 200, along with negative HER-2 expression (FISH negative). Ki-67 showed 60% of cells strongly positive stained. ER, estrogen receptor; PR, progesterone receptor; HER-2, human epidermal growth factor receptor 2. FISH, fluorescence in situ hybridization.


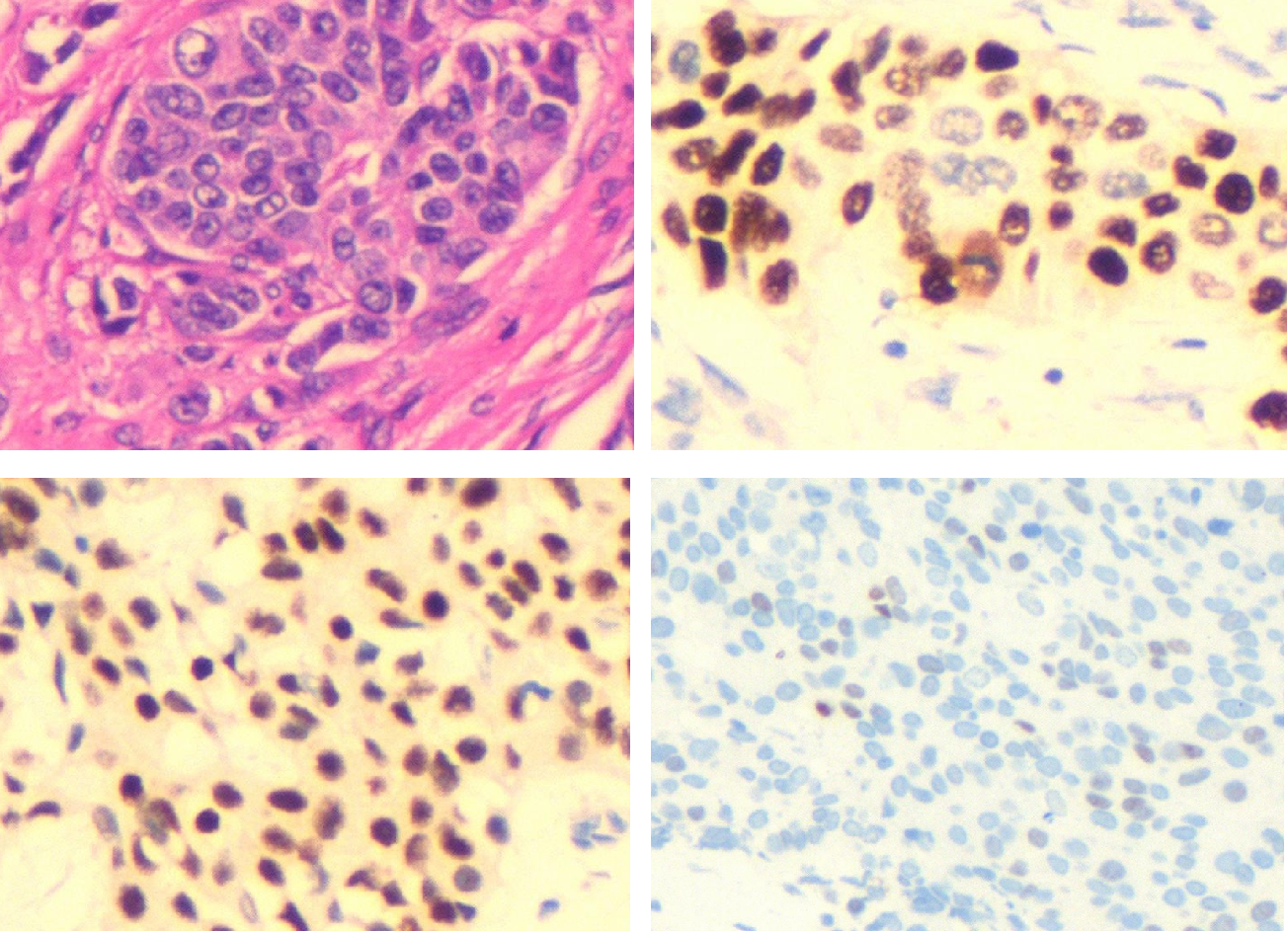


HE

ER

PR

HER-2

Figure S6. A 52-year-old patient presented with luminal A in her left breast (HE, × 400). Magnification of slides at × 400 exhibited a moderate to strong intensity with almost uniform positivity of ER (60%) and PR (80%), but a low level of HER-2 (1+). ER, estrogen receptor; PR, progesterone receptor; HER-2, human epidermal growth factor receptor 2.
